# NO_2_ inhalation promotes Alzheimer’s disease-like progression: cyclooxygenase-2-derived prostaglandin E_2_ modulation and monoacylglycerol lipase inhibition-targeted medication

**DOI:** 10.1038/srep22429

**Published:** 2016-03-01

**Authors:** Wei Yan, Yang Yun, Tingting Ku, Guangke Li, Nan Sang

**Affiliations:** 1 College of Environment and Resources, Research Center of Environment and Health, Shanxi University, Taiyuan, Shanxi 030006, PR China

## Abstract

Air pollution has been reported to be associated with increased risks of cognitive impairment and neurodegenerative diseases. Because NO_2_ is a typical primary air pollutant and an important contributor to secondary aerosols, NO_2_-induced neuronal functional abnormalities have attracted greater attention, but the available experimental evidence, modulating mechanisms, and targeting medications remain ambiguous. In this study, we exposed C57BL/6J and APP/PS1 mice to dynamic NO_2_ inhalation and found for the first time that NO_2_ inhalation caused deterioration of spatial learning and memory, aggravated amyloid β_42_ (Aβ_42_) accumulation, and promoted pathological abnormalities and cognitive defects related to Alzheimer’s disease (AD). The microarray and bioinformation data showed that the cyclooxygenase-2 (COX-2)-mediated arachidonic acid (AA) metabolism of prostaglandin E_2_ (PGE_2_) played a key role in modulating this aggravation. Furthermore, increasing endocannabinoid 2-arachidonoylglycerol (2-AG) by inhibiting monoacylglycerol lipase (MAGL) prevented PGE_2_ production, neuroinflammation-associated Aβ_42_ accumulation, and neurodegeneration, indicating a therapeutic target for relieving cognitive impairment caused by NO_2_ exposure.

Alzheimer’s disease (AD) is a common neurodegenerative disease that is becoming more prevalent in aging populations worldwide, and of the various diseases involving neurological dysfunction, AD is the only disease that has presented increased mortality over the last decade. The Alzheimer’s Association projects that unless a cure or preventative treatment is discovered, by 2050, the number of people with AD is projected to increase from more than 6 million people currently to approximately 20 million in China, from more than 5 million to approximately 16 million in the United States, and from nearly 44 million to at least 115 million worldwide^1^,^2^. The disease is characterized by the excessive deposition of β-amyloid (Aβ) plaques and neurofibrillary tangles (NFTs) in the brain, and the pathological progression is accompanied by neuroinflammation, synaptic dysfunction, progressive cognitive deficits, and memory loss associated with widespread nerve cell death^3^. Because AD is persistent, disabling and costly, the disease can severely strain health-care systems. Although the predicted increase in the frequency of AD is mainly due to population aging, other common risk factors have been proposed, including familial inheritance, ApoE polymorphisms, sex, coexistence with other disorders (such as cardiovascular diseases, brain damage, diabetes and Down syndrome), and certain environmental factors^4^,^5^. Importantly, increasing numbers of epidemiological studies have demonstrated strong links between exposure to air pollution and increased AD-related morbidity^6^,^7^,^8^,^9^.

Air pollution is resulted from a complex mixture of pollutants consisting of both particle and gas components. The identification of the specific pollutants that contribute most to health hazards could play an important role in the creation of environmental and social policies, with crucial implications for the actions taken by local authorities to reduce exposure and the risks of pollutants to public health. In this field, an important pollutant that contributes to health hazards is NO_2_, which is usually considered to constitute most of the atmospheric burden of nitrogen oxides (NOx)^10^. As reported, peak levels of up to 0.4–4 parts per million (ppm) have been encountered in the outdoors, particularly along the curbsides in downtown areas with heavy motor vehicle traffic^11^. Indoor concentrations of NO_2_ often exhibit much higher levels than those usually found outdoors, in garages, on ferries, at ice skating rinks, and in kitchens with gas ovens, NO_2_ levels can be as high as 4 ppm^12^. Therefore, NO_2_ has been confirmed as a strong indicator of outdoor and indoor air quality and a potential novel risk factor for negative health effects. In addition to traditional respiratory damage, recent epidemiological studies have linked NO_2_ pollution to an increased risk of neurological disorders, including abnormalities in general cognitive scores, on psychomotor, attention and sensory scales, and in logical memory among preschool children, middle-aged and older adults and populations exposed to occupational traffic (policemen and drivers)^13^,^14^,^15^,^16^,^17^. Whereas cognitive impairment is a clinical manifestation of many neurological diseases, it has also been termed a prodromal stage of AD that occurs in the pre-clinical phase^18^. People with mild cognitive impairment (MCI) have an increased risk of eventually developing AD or another type of dementia, with a rate of progression between 10% and 15% per year^19^. This information indicates that exposure to NO_2_ in outdoor and indoor air environments might contribute to cognitive deficits and could accelerate AD-like progression.

The real air environment is a complex system that contains various components, some of which are more plausible toxicants. Therefore, due to the different components in the mixture of air pollutants, epidemiological evidence might be insufficient to confirm the exposure-response correlation between NO_2_ and AD progression, particularly regarding the evidence from exposure markers and molecular targets. In the present study, we dynamically exposed C57BL/6J and APP/PS1 mice to NO_2_ at 2.5 and/or 5 mg/m^3^ (5 h/day, approximately one-hour average concentration every day 0.52 and 1.04 mg/m^3^), and the concentration did not exceed the permissible exposure limit of a time-weighted average concentration from National Institute for Occupational Safety and Health (NIOSH, 1.8 mg/m^3^), was about 2.17 or 4.33 times higher than the one-hour average concentration limit of national air quality secondary standards from China (0.24 mg/m^3^), and was about 2.77 or 5.53 times higher than the one-hour standard set of National Ambient Air Quality Standard (NAAQS) from the U. S. Environmental Protection Agency (EPA, approximately 0.188 mg/m^3^). Following the inhalation exposure, we found that NO_2_ air pollution poses a potential risk by promoting AD-like pathological and functional abnormalities, revealing a mechanistic basis for treating, ameliorating, or preventing AD-related outcomes in polluted areas.

## Results

### NO_2_ inhalation deteriorates spatial learning and memory and potentiates amyloid production

**Figure 1:**
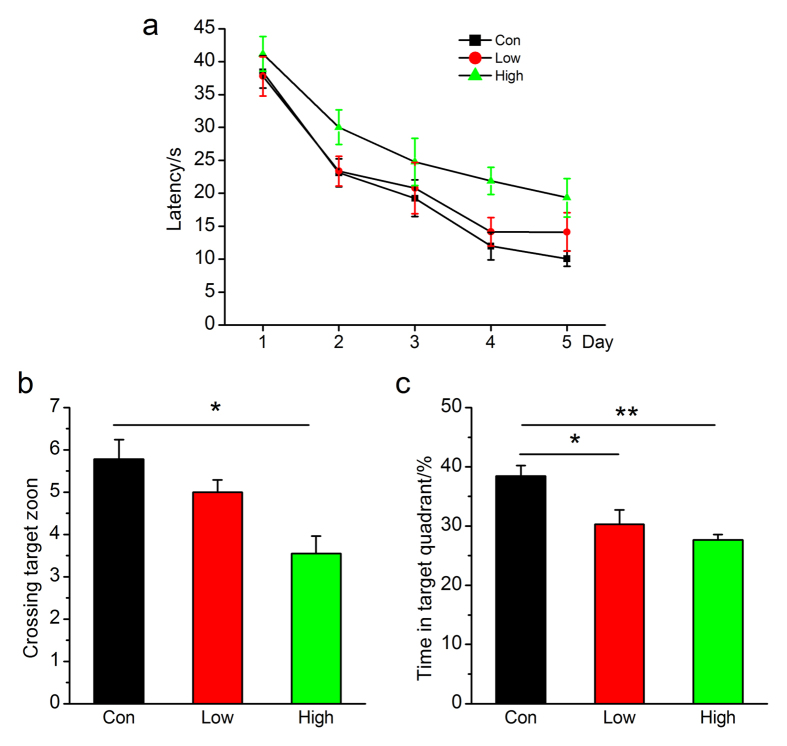
NO_2_ inhalation deteriorates spatial learning and memory in C57BL/6J mice. (**a**) Learning curve for 5 days of invisible training to find the hidden platform in the Morris water maze. Retention memory was determined using a probe trial test conducted 24 h after 5 days of training. During the probe test, the platform was removed from the pool. (**b**) Number of times crossing the target zone. (**c**) Percentages of time stayed in the target quadrant. C57BL/6J mice were exposed to 2.5 and 5 mg/m^3^ NO_2_ for 5 h/day for 4 weeks, and Morris water maze was carried out. The submerged platform was located in the center of one quadrant of the pool. Mice received visible platform training for 3 days followed by 5 days of invisible platform training. Data were expressed as means ± SE (*n* = 13 to 14 mice/group), *P < 0.05 *vs* control. Con = control; Low = 2.5 mg/m^3^ NO_2_; High = 5 mg/m^3^ NO_2_.

To determine whether NO_2_ inhalation impaired cognitive function and caused AD-like pathological abnormalities, we exposed normal C57BL/6 J mice to 2.5 and 5.0 mg/m^3^ NO_2_ via dynamic inhalation. For the first time, we observed changes in spatial learning and memory: NO_2_ inhalation caused dose-dependent impairment of mouse performance on the Morris water maze test, and compared to control animals, NO_2_-exposed mice required a longer time to find the hidden platform (Fig. 1a). In a probe trial, the number of mice crossing the target zone and the time the mice stayed in the target quadrant were reduced in a dose-dependent manner, and a significant difference was observed after inhalation of 5 mg/m^3^ NO_2_ (Fig. 1b,c).

**Figure 2:**
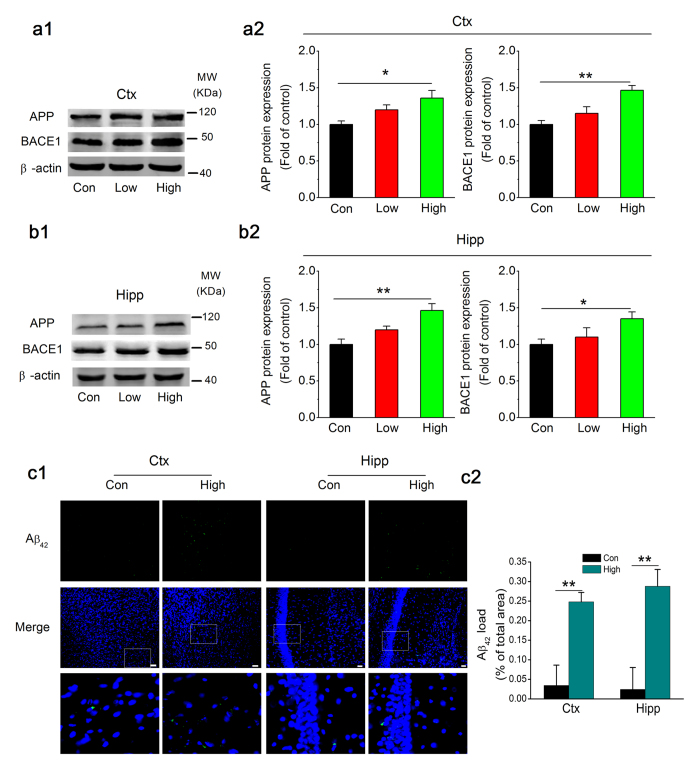
NO_2_ inhalation potentiates amyloid production in C57BL/6J mice. (**a**) Immunoblot analysis of APP and BACE1 expression in the cortex of C57BL/6J mice before and after NO_2_ inhalation exposure. (**b**) Immunoblot analysis of APP and BACE1 in the hippocampus of C57BL/6J mice before and after NO_2_ inhalation exposure. C57BL/6J mice were exposed to 2.5 and 5 mg/m^3^ NO_2_ for 5 h/day for 4 weeks, and immunoblot analysis of APP and BACE1 were determined. Data were presented as means ± SE (*n* = 6 to 8 mice/group), *P < 0.05 and **P < 0.01 *vs* control. Blots were cropped in this figure, but the gels were run under the same experimental conditions, and full-length blots are presented in Supplementary Figure S1. (**c**) Aβ_42_ deposition (Aβ_42_: green, DAPI: blue) in the cortex and hippocampus of C57BL/6J mice exposed to air and 5 mg/m^3^ NO_2_ (*n* = 2, data were averaged from 5 to 6 sections per mice). Scale bars = 25 μm. Con = control; Low = 2.5 mg/m^3^ NO_2_; High = 5 mg/m^3^ NO_2_.

Progressive memory loss and cognitive decline are believed to be promoted by Aβ-induced damage, which plays a major causative role in AD pathogenesis^3^. Aβ peptides are derived from the sequential cleavage of amyloid precursor protein (APP), and the enzymatic hydrolysis is primarily mediated by β-site APP cleaving enzyme 1 (BACE1). Among a diverse group of Aβ peptides that vary in length depending on the specific cleavage site, the 42-residue-long form (Aβ_42_) is the most pathogenic structure^20^. To provide pathological evidence of the deterioration of spatial learning and memory due to NO_2_, we further detected the expression of proteins related to Aβ synthesis and the deposition of Aβ_42_. As shown in Fig. 2a and b, inhalation of 5 mg/m^3^ NO_2_ slightly but significantly elevated APP and BACE1 expression in both the cortex and the hippocampus, and it selectively caused Aβ_42_ deposition (see Fig. 2c; significant accumulation of Aβ_42_ in 2 animals were observed based on 5 animals in response to 5 mg/m^3^ NO_2_ inhalation). These results indicated that NO_2_ inhalation could potentiate Aβ_42_ production and contribute to the deterioration of spatial learning and memory.

### NO_2_ inhalation aggravates cognitive deficits and amyloid deposition in APP/PS1 mice

**Figure 3:**
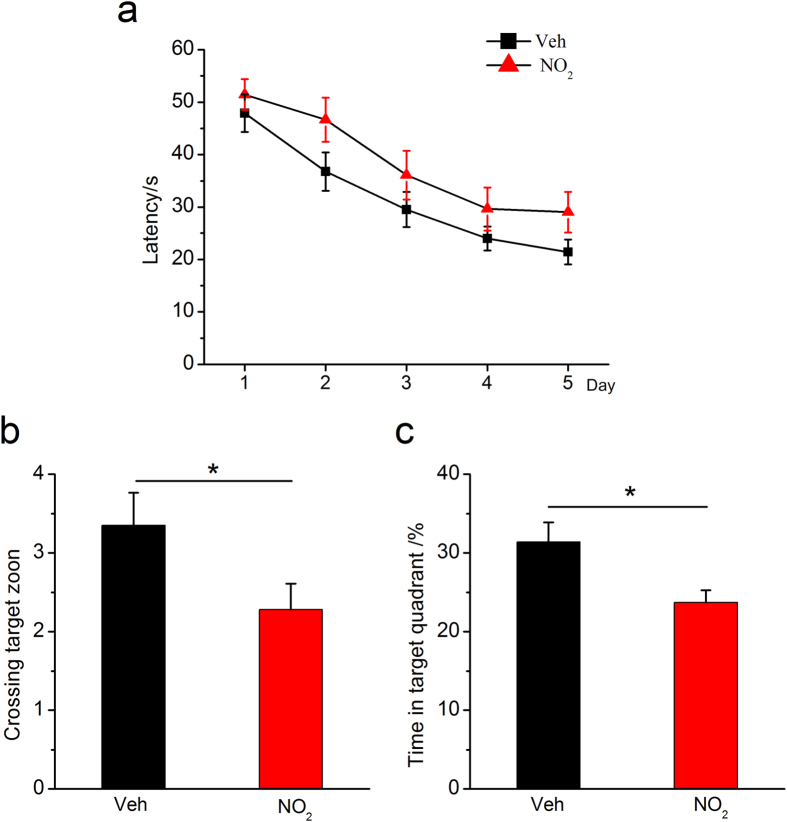
NO_2_ inhalation aggravates spatial learning and memory deterioration in APP/PS1 mice. (**a**) Learning curve for 5 days of invisible training to find the hidden platform in the Morris water maze. Retention memory was determined using a probe trial test conducted 24 h after 5 days of training. During the probe test, the platform was removed from the pool. (**b**) Number of times crossing the target zone. (**c**) Percentages of time stayed in the target quadrant. APP/PS1 mice were exposed to 5 mg/m^3^ NO_2_ for 5 h/day for 4 weeks, and Morris water maze was carried out. The submerged platform was located in the center of one quadrant of the pool. Mice received visible platform training for 3 days followed by 5 days of invisible platform training. Data were expressed as means ± SE (*n* = 12 to 14 mice/group), *P < 0.05 *vs* vehicle control (APP/PS1 mice exposed to room air). Veh = vehicle; NO_2_ = 5 mg/m^3^ NO_2_.

**Figure 4:**
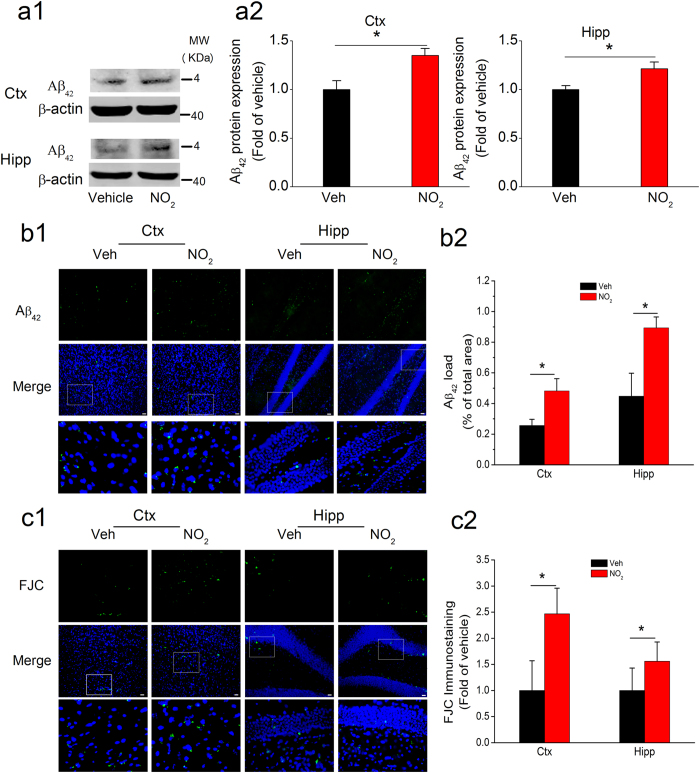
NO_2_ inhalation exacerbates amyloid deposition and neurodegeneration in APP/PS1 mice. (**a**) Immunoblot analysis of Aβ_42_ in the cortex and hippocampus of APP/PS1 mice before and after NO_2_ inhalation exposure. APP/PS1 mice were exposed to 5 mg/m^3^ NO_2_ for 5 h/day for 4 weeks, and immunoblot analysis of Aβ_42_ was determined. Data were expressed as means ± SE (n = 5 to 6 mice/group), *P < 0.05 *vs* vehicle control (APP/PS1 mice exposed to room air). Blots were cropped in this figure, but the gels were run under the same experimental conditions, and full-length blots are presented in Supplementary Figure S2. (**b**) Aβ_42_ deposition (Aβ_42_: green, DAPI: blue) in the cortex and hippocampus of APP/PS1 mice before and after NO_2_ inhalation exposure. (**c**) Degenerated neurons (FJC, a neurodegeneration marker, green, DAPI: blue) in the cortex and hippocampus of APP/PS1 mice before and after NO_2_ inhalation exposure. Data were averaged from 4 sections per animal (*n* = 5 mice/group). Scale bars = 25 μm. Veh = vehicle; NO_2_ = 5 mg/m^3^ NO_2_.

If the above indications were correct, there should be a link between NO_2_ inhalation and AD progression. Therefore, NO_2_ inhalation should aggravate the pathological abnormalities and cognitive deficits of APP/PS1 transgenic mice. Considering that APP/PS1 animals develop amyloid plaques beginning at the age of 5–6 months^21^, to test this hypothesis, we treated 5-month-old APP/PS1 transgenic mice with NO_2_ (5 mg/m^3^) for 4 weeks, followed by an investigation of their spatial learning and memory and related pathological alterations. As shown in Fig. 3, NO_2_-exposed animals exhibited longer latency, fewer crossings of the target zone, and a shorter time in the target quadrant. Importantly, we observed significant aggravation of Aβ_42_ accumulation and deposition in the cortical and hippocampal regions after NO_2_ inhalation (Fig. 4a,b). Because neurodegenerative change is an important feature of the neuropathology of MCI and AD, we further assessed whether NO_2_ inhalation could exacerbate neurodegeneration in the AD model mice. The results indicated that NO_2_ exposure significantly increased the number of FJC-positive neurons (Fig. 4c), which substantiated the notion that NO_2_ inhalation aggravated Aβ deposition, worsened cognitive deficits, and contributed to AD progression.

### Cyclooxygenase-2 (COX-2)-mediated arachidonic acid metabolism participates in NO_2_ aggravation of AD progression

**Figure 5:**
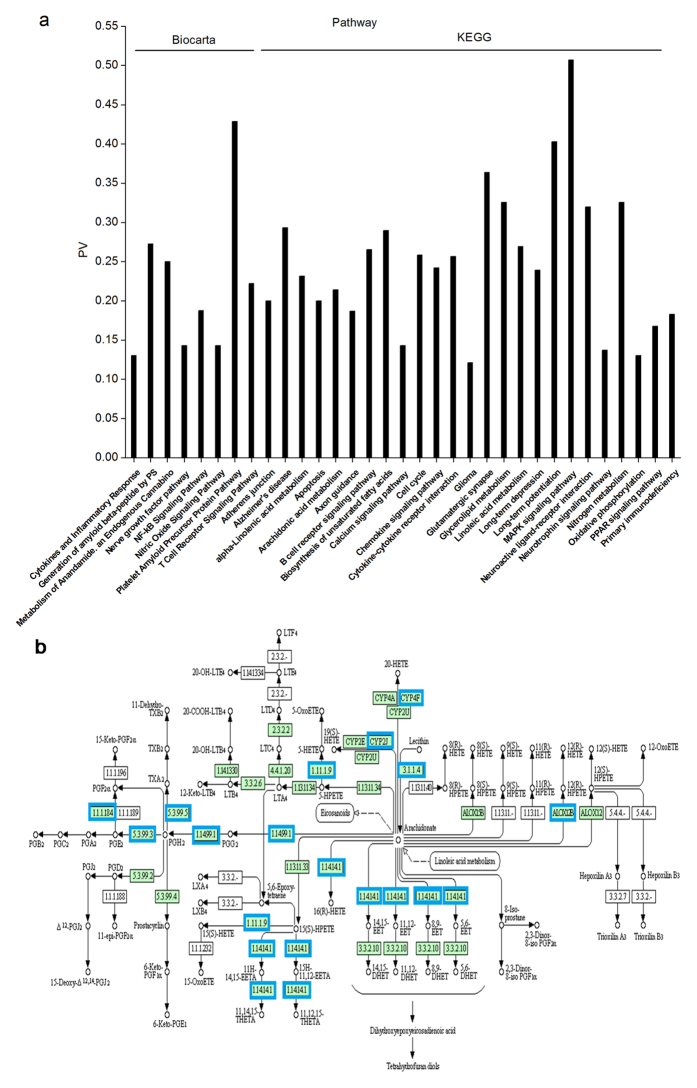
Arachidonic acid (AA) metabolism participates in the aggravation of AD-like pathology following NO_2_ inhalation. (**a**) Pathway analysis of genes associated with differentially expressed genes (DEGs) in the hippocampus of APP/PS1 mice in response to NO_2_ inhalation (*n* = 4 mice/group). Genes statistically changed after NO_2_ inhalation mostly pointed to the synaptic plasticity and learning and memory function, including long-term potentiation/depression (LTP/D), glutamatergic synapse, neurotrophin signaling and calcium signaling pathway. The generation of amyloid β-peptide by PS1, platelet amyloid precursor protein pathway and Alzheimer’s disease, revealed Aβ peptide production and deposition-derived AD pathogenesis after NO_2_ treatment. Additionally, AA metabolism in KEGG pathway analysis was observed. PV means percentage value of the number of related DEGs to the number of total related genes to a category. The significantly impacted categories shown were defined by limitation of *P* value (*P* < 0.01). Note: Only items of interest and related to neurobiological processes were presented due to the large size of the figure. (**b**) DEGs in AA metabolism pathway by Pathway-Express analysis. The genes statistically changed (*P* < 0.01) were indicated in blue borders, and the genes unchanged were marked with green gene boxes.

To clarify the potential modulatory mechanisms by which NO_2_ inhalation exacerbates AD progression, gene chip analysis was performed in APP/PS1 mouse brains before and after NO_2_ exposure. In total, 5,487 significantly differentially expressed genes were identified (*P* < 0.01). Then, we investigated the regulatory potential of these target genes in the Kyoto Encyclopedia of Genes and Genomes (KEGG) and Biocarta pathways; the items associated with neurological disorders are presented in Fig. 5a. These results indicated that the genes that significantly changed after NO_2_ inhalation are mostly involved in synaptic function and learning and memory abilities, including long-term potentiation/depression (LTP/D), the nitric oxide signaling pathway, glutamatergic synapses, neurotrophin signaling, and the calcium signaling pathway. Additionally, the generation of Aβ peptide by PS1, a platelet amyloid pathway protein and an early indicator of AD, revealed Aβ peptide production and deposition-derived AD pathogenesis after NO_2_ treatment. These results provided further evidence that NO_2_ inhalation contributed to the deterioration of spatial learning and memory and potentiated AD-like amyloid pathology.

**Figure 6:**
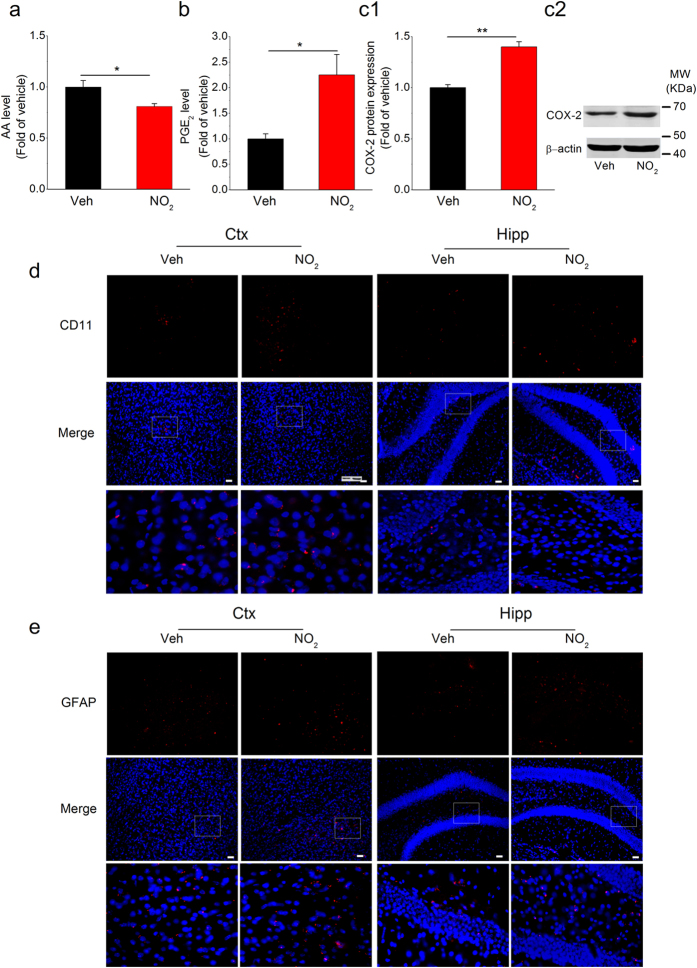
NO_2_ inhalation promotes COX-2 elevation-mediated PGE_2_ production and following neuroinflammation in APP/PS1 mice. APP/PS1 mice were exposed to 5 mg/m^3^ NO_2_ for 5 h/day for 4 weeks, and AA (**a**) and PGE_2_ (**b**) contents and COX-2 expression (**c**) in the cortex were determined. Data were expressed as means ± SE (*n* = 8 mice/group), *P < 0.05 and **P < 0.01 *vs* vehicle control (APP/PS1 mice exposed to room air). Blots were cropped in this figure, but the gels were run under the same experimental conditions, and full-length blots are presented in Supplementary Figure S3. Also, reactive microglial cells (CD11b/OX42, a microglial marker, red) (**d**) and activated astrocytes (GFAP, an astrocytic marker, red) (**e**) in the cortex and hippocampus of APP/PS1 mice in response to NO_2_ were detected. Scale bars = 25 μm. Veh = vehicle; NO_2_ = 5 mg/m^3^ NO_2_.

These phenomena led to a crucial issue pertaining to the molecular regulatory mechanism involved in these effects. Intriguingly, the KEGG pathway analysis revealed the involvement of arachidonic acid (AA) metabolism. AA-derived prostaglandin E_2_ (PGE_2_), which arises mainly from the cyclooxygenase-2 (COX-2) reaction, is an important mediator that stimulates Aβ formation, promotes neuroinflammatory responses, and modulates synaptic events^22^,^23^. Therefore, it appears that COX-2-mediated AA metabolism is a possible mechanism by which NO_2_ promotes AD progression. The genes in the AA metabolism pathway that significantly changed are presented in Fig. 5b and Table 1. Among these genes, *ptgs2* (also known as *cox-2*) was up-regulated after NO_2_ inhalation (1.26- fold of control levels) suggesting the involvement of COX-2-derived PGE_2_ synthesis. To provide additional evidence for this relationship, we determined the levels of AA and PGE_2_ and the COX-2 expression in the cortex. As shown in Fig. 6a–c, the principal metabolic effects after NO_2_ inhalation were a reduction in AA levels and an increase in PGE_2_ production accompanied by significant COX-2 elevation. COX-2-mediated production of PGE_2_ is a known inflammatory mediator that enhances the neuroinflammatory response, which is involved in the complex cascade and contributes to synaptic and cognitive deficits in AD^24^. Subsequently, we used specific markers (OX42/CD11b and GFAP) in APP/PS1 animals to determine the activation of microglia and reaction of astrocytes before and after NO_2_ exposure. As shown in Fig. 6d and e, obvious increases in CD11b and GFAP expression occurred in the cortex and hippocampus, indicating amplified neuroinflammation following NO_2_ inhalation. These findings indicated that COX-2-mediated conversion of AA into PGE_2_ played a key role in NO_2_-mediated aggravation of AD progression.

### MAGL disruption suppresses COX-2-derived PGE_2_ and restores AD-like pathology and cognitive deterioration in response to NO_2_ inhalation

**Figure 7:**
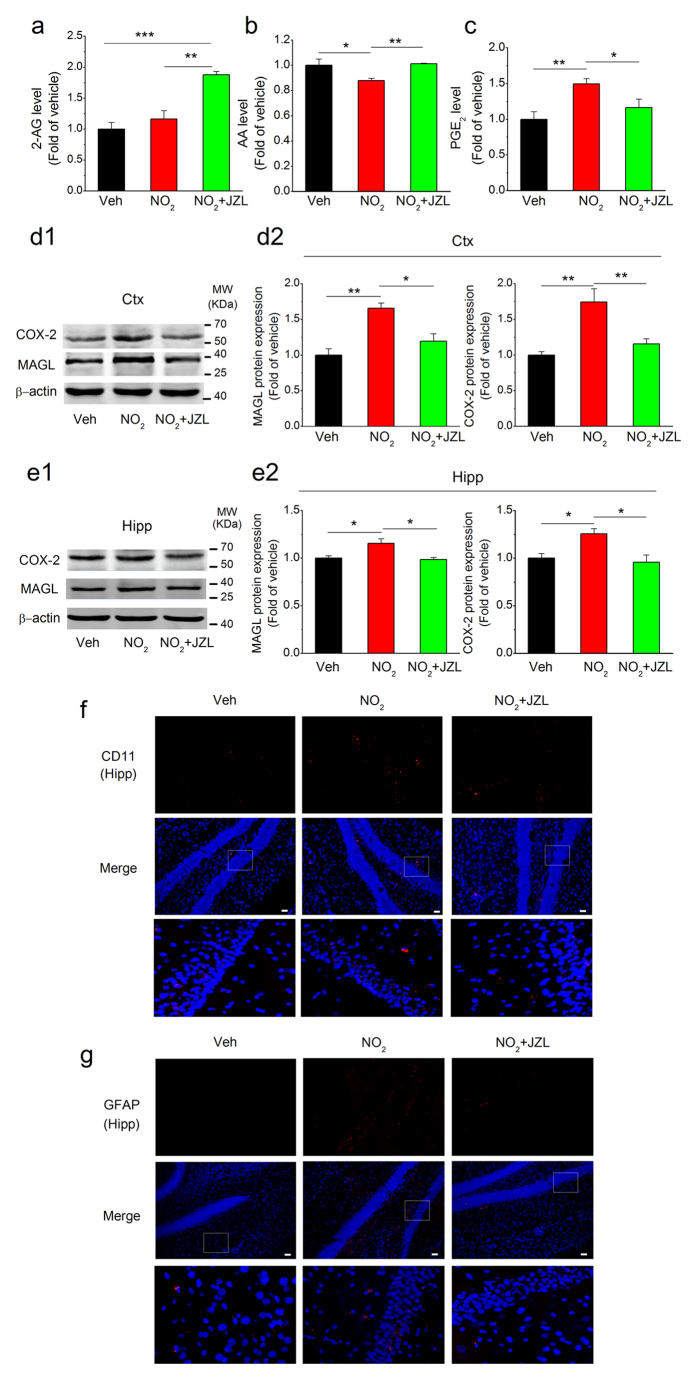
MAGL inhibition suppresses COX-2-derived PGE_2_ generation and neuroinflammation following NO_2_ inhalation in C57BL/6J mice. C57BL/6J mice were exposed to 5 mg/m^3^ NO_2_ for 5 h/day for 4 weeks in the absence or presence of MAGL inhibitor, JZL184 (8 mg/kg, i.p.), and the levels of 2-AG (**a**), AA (**b**) and PGE_2_ (**c**) in the cortex were determined. Also, the expression of COX-2 and MAGL in the cortex (**d**) and hippocampus (**e**) were quantificated. Data were expressed as means ± SE (*n* = 6 to 8 mice/group), *P < 0.05 and **P < 0.01 *vs* vehicle control group or NO_2_ treatment group. Blots were cropped in this figure, but the gels were run under the same experimental conditions, and full-length blots are presented in Supplementary Figure S4. (**f**) Reactive microglial cells (CD11b/OX42, a microglial marker, red) in the hippocampus of C57BL/6J mice. (**g**) Activated astrocytes (GFAP, an astrocytic marker, red) in the hippocampus of C57BL/6J mice. Scale bars = 25 μm. Veh = vehicle; NO_2_ = 5 mg/m^3^ NO_2_; NO_2_ + JZL = 5 mg/m^3^ NO_2_ + 8 mg/kg JZL184.

In this manner, if the COX-2-mediated conversion of AA into PGE_2_ modulates NO_2_-induced neuronal dysfunction, COX-2 inhibition should be the most effective method for relieving these injuries. However, the occurrence of serious side effects, such as the gastrointestinal toxicity of nonselective COX-2 inhibitors and the cardiovascular risk caused by selective COX-2 inhibitors, has limited their translational potential for neuroinflammatory syndromes^25^ and has promoted the search for novel medications. To address this problem, the experimental findings that 2-arachidonoylglycerol (2-AG) protects neurons from inflammation- and neurodegeneration-induced damage has attracted our focus to endocannabinoids (eCBs). A growing body of evidence indicating the antioxidant, anti-inflammatory and neuroprotective properties of eCBs has recently accumulated^26^,^27^,^28^,^29^. Although still preliminary, the modulation of eCBs could constitute an innovative therapeutic approach for the treatment of AD. As the most abundant endocannabinoid in the brain, 2-AG is produced mainly from diacylglycerol (DAG) by diacylglycerol (DGL) lipase, and it is hydrolyzed to AA by monoacylglycerol lipase (MAGL). MAGL plays a major role in the regulation of 2-AG levels, and its inhibition elevates endogenous 2-AG and decreases the production of AA and downstream AA-derived eicosanoids. Importantly, endogenous 2-AG also suppresses the excessive expression of COX-2^30^. This information was revealed by the available medications for neuroinflammatory and neurodegenerative diseases, and MAGL is considered to be a potential target. Therefore, we hypothesized that strengthening endogenous 2-AG by inhibiting MAGL could prevent neuronal insults and relieve the subsequent NO_2_ inhalation-induced cognitive deficits by limiting COX-2-derived PGE_2_ production. To test this hypothesis, we exposed C57BL/6 J mice to 5 mg/m^3^ NO_2_ in the absence or presence of pre-treatment with JZL184, a highly selective and potent MAGL inhibitor, and we detected the AA and PGE_2_ levels, COX-2 expression, and neuroinflammation in the brain. The results indicated that NO_2_ inhalation elevated MAGL and COX-2 expression and caused significant AA reduction, PGE_2_ elevation, and obvious activation of astrocytes and microglia. However, the chemical exposure did not alter 2-AG content (see Fig. 7a). Importantly, pre-treatment with the MAGL inhibitor JZL184 significantly augmented the levels of endogenous 2-AG and AA, attenuated PGE_2_ release, and alleviated neuroinflammation in the brain following NO_2_ inhalation. These findings indicated that increasing the levels of the endocannabinoid 2-AG by disrupting MAGL prevented the COX-2-derived conversion of AA into PGE_2_ and neuroinflammation resulting from NO_2_ exposure.

**Figure 8:**
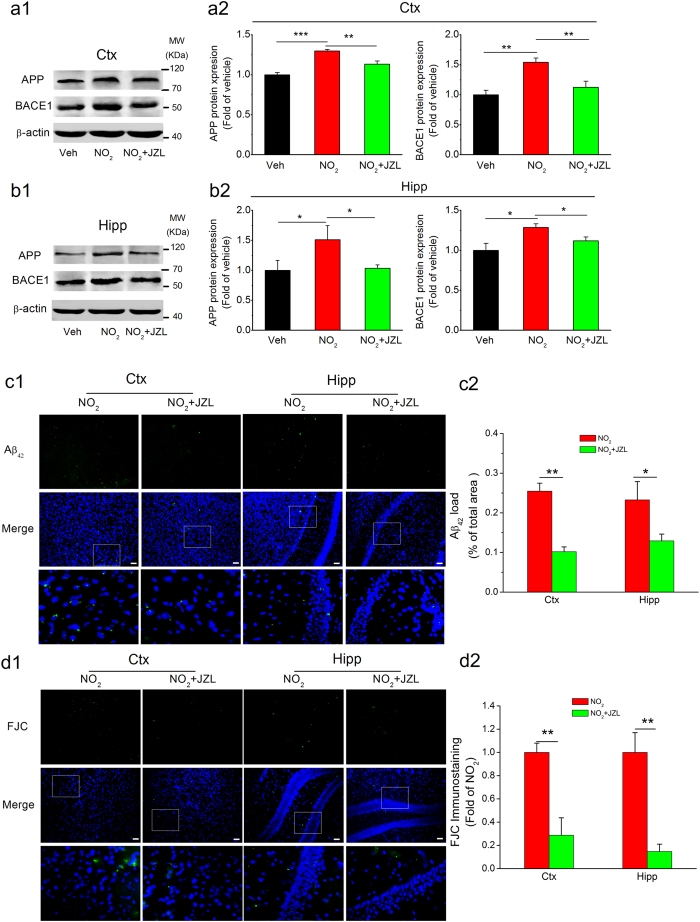
MAGL inhibition represses Aβ accumulation and neurodegeneration in response to NO_2_ inhalation in C57BL/6J mice. C57BL/6J mice were exposed to 5 mg/m^3^ NO_2_ for 5 h/day for 4 weeks in the absence or presence of MAGL inhibitor, JZL184 (8 mg/kg, i.p.), and the expression of APP and BACE1 in the cortex (**a**) and hippocampus (**b**) were determined. Data were expressed as means ± SE (*n* = 6 to 8 mice/group), *P < 0.05, **P < 0.01 *vs* vehicle control group or NO_2_ treatment group. Blots were cropped in this figure, but the gels were run under the same experimental conditions, and full-length blots are presented in Supplementary Figure S5. (**c**) Accumulation and deposition of Aβ_42_ (green) in the brain. (**d**) Degenerated neurons (FJC, a neurodegeneration marker, green) in the cortex and hippocampus of mice. Scale bars = 25 μm. Data were averaged from 4 sections per mice, and *n* = 4 to 5 mice/group; *P < 0.05, **P < 0.01 *vs* NO_2_ treatment group. Veh = vehicle; NO_2_ = 5 mg/m^3^ NO_2_; NO_2_ + JZL = 5 mg/m^3^ NO_2_ + 8 mg/kg JZL184.

**Figure 9:**
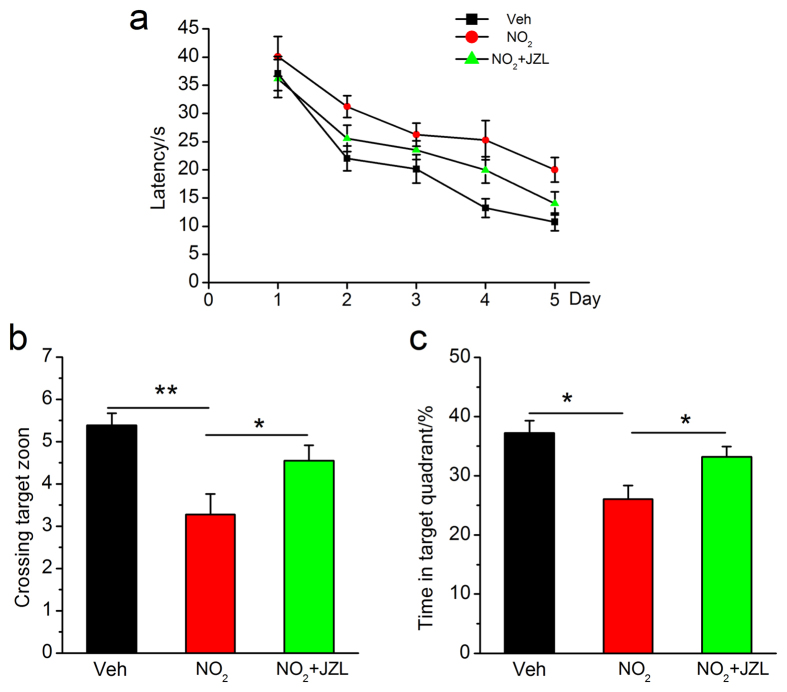
MAGL disruption restores cognitive deterioration in response to NO_2_ inhalation in C57BL/6J mice. (**a**) Learning curve for 5 days of invisible training to find the hidden platform. (**b**) Number of times crossing the target zone. (**c**) Percentages of time stayed in the target quadrant. C57BL/6J mice were exposed to 5 mg/m^3^ NO_2_ for 5 h/day for 4 weeks in the absence or presence of MAGL inhibitor, JZL184 (8 mg/kg, i.p.), and Morris water maze was carried out. Data were expressed as means ± SE (*n* = 12 to 14 mice/group), *P < 0.05, **P < 0.01 *vs* vehicle control group or NO_2_ treatment group. Veh = vehicle; NO_2_ = 5 mg/m^3^ NO_2_; NO_2_ + JZL = 5 mg/m^3^ NO_2_ + 8 mg/kg JZL184.

If COX-2-derived PGE_2_ signaling modulates the NO_2_-mediated aggravation of AD progression and if MAGL inhibition prevents the PGE_2_ release after the COX-2-mediated conversion of AA, MAGL disruption should suppress Aβ accumulation and reverse cognitive deterioration following exposure to NO_2_. Following this logic, we further determined the alterations in Aβ_42_ accumulation and neurodegeneration. As presented in Fig. 8, JZL184 significantly reduced NO_2_-induced APP and BACE1 elevation, Aβ_42_ deposition, and neurodegeneration. To determine the functional outcomes of APP and BACE1 elevation in response to MAGL inhibition, we detected the changes in cognitive function and found that JZL184 also significantly reversed the NO_2_-induced impairment of spatial learning and memory (Fig. 9). Reports have shown that PGE_2_ could significantly elevate APP expression by activating EP2/EP4 receptors, thus leading to the accumulation of Aβ^31^. Therefore, these results indicated that the elevation of endogenous 2-AG levels via MAGL disruption prevented COX-2-mediated PGE_2_ production, reduced the neuroinflammation-associated accumulation of Aβ_42_, and ameliorated subsequent cognitive impairment in response to NO_2_ inhalation.

## Discussion

AD has become a major public health concern as the world’s population ages. It has been estimated that in 2015, there were more than 40 million persons worldwide living with dementia and the global incidence of AD is expected to double in the next 20 years^2^. Emerging evidence has indicated that air pollution elevates markers of brain inflammation and contributes to the risk of Alzheimer’s-type neurodegenerative disease. NO_2_ is an important air pollutant arising mainly from anthropogenic emissions, and the global NO_2_ map measured by Aura’s Ozone Monitoring Instrument (OMI) has indicated that Asia has been among the noticeable hot spots of pollution in the last decade due to population increases and the industrialization of Asian countries^32^. Unfortunately, by the middle of the century, the Asia Pacific region will have more than half of the total number of people with dementia worldwide^33^. Because NO_2_ inhalation promotes AD-like pathologies and cognitive deficits, including AD progression, there is a pressing need to understand the molecular ways in which AD develops in NO_2_-polluted areas. This understanding could be a critical step in identifying biomarkers that predict future clinical symptoms and can be used as outcome measurements in clinical trials of disease-modifying agents targeted at efficiently halting or reversing neurological disorders.

Here, we found for the first time that NO_2_ inhalation causes the deterioration of spatial learning and memory and tends to promote Aβ deposition in a concentration-dependent manner. Importantly, NO_2_ aggravated cognitive impairment, amyloid deposition, neuroinflammation and neurodegeneration in APP/PS1 model mice. AD is characterized by progressive neurodegeneration, and neurofibrillary degeneration and neuroinflammation are prominent features of the disease^3^. Early versions of the amyloid cascade hypothesis of AD posited that Aβ peptide deposition triggered inflammation, tau hyperphosphorylation, synapse damage and neuronal loss, which were considered causal of cognitive decline^34^. Our findings confirmed the relationships between air NO_2_ exposure and the neuropathological responses typically associated with AD *in vivo.*

Air pollution is a multifaceted toxic chemical mixture capable of assaulting the central nervous system (CNS). A coherent pathway linking exposure to air pollution and brain damage includes a chronic inflammatory process involving the respiratory tract, which results in a systemic inflammatory response with the production of inflammatory mediators capable of reaching the brain; continuous expression of crucial inflammatory mediators in the CNS at low levels; and the formation of oxidative species (ROS)^35^. Considering our previous study that acute NO_2_ inhalation induces the release of proinflammatory elements, including tumor necrosis factor-α (TNF-α), interleukin-1β (Il-1β) levels and intercellular adhesion molecule 1 (ICAM-1) in rat lung and heart, causing the elevation of reactive ROS and induced excessive expression of cytokines TNF-α and Il-1β in rat cortex^36^,^37^,^38^, we speculated that NO_2_ exposure may causes pro-inflammatory signals originating in peripheral tissues/organs including the lung and the cardiovascular system, giving rise to a systemic-induced cytokine response that transfers inflammation to the brain. However, the specific molecular mechanism regulated NO_2_-induced neuronal insults has remained unclear. To address this issue, we first performed a whole gene sequence analysis in APP/PS1 animals before and after NO_2_ exposure. Based on the genes that were significantly changed, KEGG and Biocarta pathway graphing not only confirmed AD pathology and cognitive impairment, but also suggested a potential modulating process. COXs are the key rate-limiting enzymes that determine the first two steps in the biosynthesis of PGs from AA. Importantly, COX-2-mediated PGE_2_ production is initiated by an ROS attack and has been implicated in synaptic signaling and certain neurologic abnormalities, such as traumatic brain injury, stroke, epilepsy and AD^39^. Therefore, AA metabolism and the elevation of *ptgs2*, which catalyze the synthesis of PGE_2_, attracted our attention. Interestingly, we found COX-2 elevation, AA reduction, excessive PGE_2_ production and subsequent neuroinflammation in APP/PS1 mice after NO_2_ exposure, and we confirmed these results in APP/PS1 and C57BL/6 J mice. These findings indicated that the COX-2-derived conversion of AA into PGE_2_ might be a key modulator in NO_2_-mediated aggravation of AD progression.

The human epidemiological literature has shown elevated levels of PGE_2_ in the cerebrospinal fluid of early-stage AD patients^40^. Additionally, research performed in AD model mice has shown that individual PGE_2_ G protein-coupled receptors contribute to the development of AD, and global deletion of these receptors reduces amyloid pathology^41^. By contrast, Aβ is produced by proteolysis of APP by β- and γ-secretases and characterizes AD pathogenesis. Previous *in vivo* and *in vitro* experiments have indicated that PGE_2_ release stimulates APP expression via EP2/EP4 receptors in parallel with Aβ deposition, ultimately resulting in AD pathogenesis^31^. Consistent with these results, our present data showed that APP and BACE1 expression and Aβ_42_ deposition were significantly augmented in response to COX-2 over-expression and PGE_2_ elevation following NO_2_ inhalation. Moreover, NO_2_ inhalation exacerbated the weakening of spatial learning and memory in APP/PS1 mice. It has been reported that NO_2_ is a typical indicator of traffic-related atmospheric pollution, and the increasing severity and duration of traffic congestion have greatly increased NO_2_ emissions. In addition, several studies have shown that traffic exhaust might be neurotoxic and could be expected to promote CNS dysfunction, which could manifest as cognitive deficits^42^. Reports from Mexico City have revealed that serious air pollution stimulates nuclear factor-κB activation and nitric oxide generation contributing to the increased release of the pro-inflammatory markers IL-1β and TNF-a in animal brains. Moreover, pollution has been associated with COX-2 up-regulation and Aβ_42_ deposition in human autopsies^43^,^7^. Consistent with these results from previous studies, our present findings provided further evidence that the COX-2-catalyzed production of PGE_2_ participated in NO_2_-induced AD-like neurological dysfunction, indicating that COX-2-mediated PGE_2_ generation could be used as a predictive marker of the clinical symptoms associated with cognitive dysfunction in NO_2_-exposed individuals.

Based on this information, the next critical step was to determine the pathways that can be efficiently targeted to halt or reverse these neurological outcomes. Non-steroidal anti-inflammatory drugs (NSAIDs) are effectively reducing neurotoxicity and have been associated with neuronal protection. The best-characterized actions of NSAIDs are their anti-inflammatory properties, which are exerted primarily through their irreversible inactivation of the COX enzyme to limit the production of pro-inflammatory PGs. In particular, previous studies have indicated that COX-2-selective inhibitors, a typical type of NSAIDs, suppress COX-2-derived PGE_2_ signaling and prevent neuronal insults and AD development^44^. Based on these findings, COX-2-selective inhibitors, including SC-58125, celecoxib, and NS398, should target NO_2_-induced PGE_2_ signaling and neurological dysfunction. Because these medications are already available, they could potentially be used to prevent and treat injuries in polluted areas. However, COX-2-selective inhibitors can also increase blood pressure, induce or worsen cardiac failure, and impair kidney function to the point of renal failure even though these inhibitors do not induce the gastric ulcerations associated with the use of traditional NSAIDs^45^. These unexpected side effects have limited their medical use in certain markets. Therefore, we must find novel approaches for inhibiting COX-2-derived PGE_2_ signaling and to relieve AD-like progression following NO_2_ inhalation. The eCB system, which acts on demand to confer neuroprotection, has received a fair amount of attention over the past few years. Among the identified eCBs, 2-AG is bioactive and abundant in the brain that is well known for its significant functions in synaptic modification, the resolution of neuroinflammation and neuronal survival^30^,^46^. Importantly, the anti-inflammatory and neuroprotective effects of 2-AG appear to be realized by suppressing the activity and expression of COX-2 that occur in response to harmful insults^47^. This information suggests that strengthening 2-AG signaling might be a safe and effective way to limit the COX-2-derived PGE_2_ pathway following NO_2_ exposure. As one of the most studied eCBs, 2-AG is mainly synthesized from DAG by DGL and is hydrolyzed to AA by MAGL. MAGL is responsible for approximately 85% of brain 2-AG hydrolytic activity, and MAGL disruption enhances the endogenous levels of 2-AG and provides beneficial effects in resolving ongoing inflammation in the brain, which is the root of many neurodegenerative diseases^48^. As such, in the present study, we chose to investigate whether MAGL inhibition had neuroprotective effects on NO_2_-induced AD-like symptoms.

Here, we raised endogenous 2-AG levels by applying the selective MAGL inhibitor JZL184, providing convincing evidence that MAGL inhibition elevates brain 2-AG levels and blocks AA metabolism by suppressing COX-2 expression and PGE_2_ release. These findings suggested that the increased levels of endogenous 2-AG resulting from MAGL inhibition were sufficient to restore the negative neurological effects caused by NO_2_. Additionally, the NO_2_-mediated elevation of APP expression and PGE_2_-stimulated Aβ_42_ production were significantly reduced by endogenous 2-AG-induced inhibition of COX-2. Aβ is the initiator of AD pathogenesis, which is also prominently featured in neuroinflammation that occurs in association with activated microglia and reactive astrocytes, neuronal degeneration, and synaptic dysfunction^49^. In the present study, NO_2_ inhalation destroyed the functional normality of synaptic transmission by altering the glutamatergic synaptic and LTP/D pathway, enhancing microglial and astrocyte activation and increasing neuronal degeneration, ultimately causing learning and memory disorders. Importantly, MAGL inhibition relieved the NO_2_-induced aggravation of the classical neuropathological changes related to AD *in vivo*, including the attenuation of neuroinflammation and neurodegeneration and the restoration of spatial learning and memory. Thus, we propose that elevating endogenous 2-AG by inactivating MAGL has beneficial effects on suppressing COX-2-derived PGE_2_ signaling and thereby contributes to treating, ameliorating, or preventing AD-like progression in NO_2_-polluted areas.

**Figure 10:**
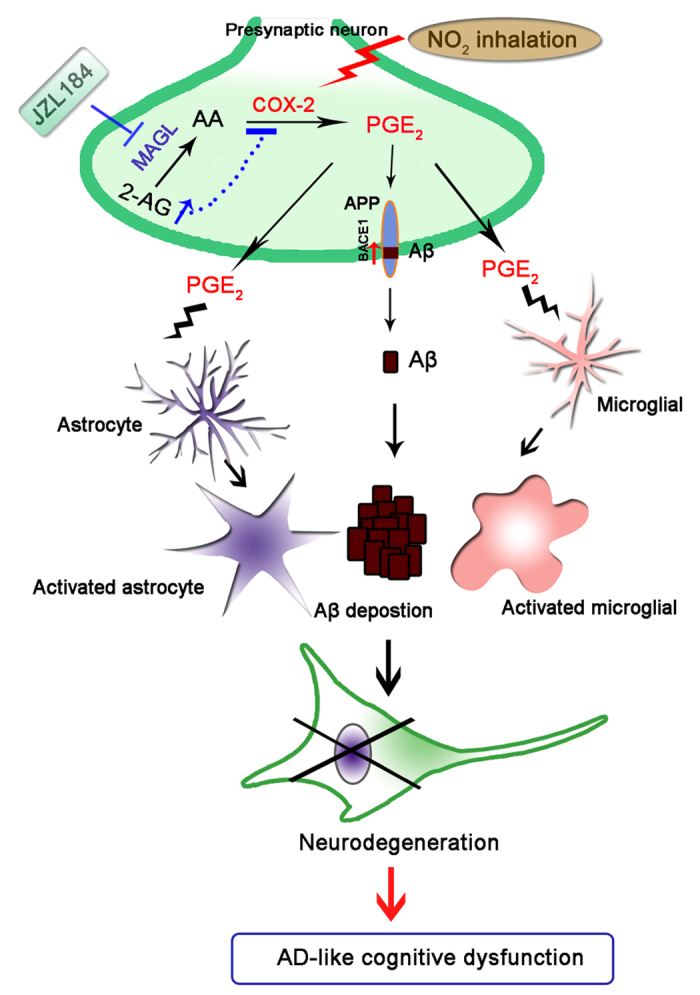
A hypothetical cartoon illustrating that NO_2_ inhalation promotes AD-like progression: COX-2-derived PGE_2_ modulation and MAGL inhibition-targeted medication. NO_2_ inhalation deteriorates spatial learning and memory, potentiates Aβ production and contributes to AD progression, and this action appears to be mediated by COX-2-derived AA metabolism to PGE_2_. Furthermore, strengthening endogenous 2-AG level by MAGL disruption effectively inhibits excessive PGE_2_ release-regualted neuroinflammation and Aβ accumalation, and finally restores impaired spatial learning and memory in response to NO_2_ inhalation.

In summary, NO_2_ inhalation deteriorated spatial learning and memory, potentiated Aβ production, and contributed to AD progression. These actions appear to be mediated by the COX-2-derived AA metabolism of PGE_2_. Furthermore, strengthening the endogenous 2-AG levels via MAGL disruption effectively inhibited the neuroinflammation and Aβ accumulation induced by the excessive release of PGE_2_, thereby restoring impaired spatial learning and memory in response to NO_2_ inhalation (Fig. 10). These findings emphasized the potential risks of NO_2_ in indoor and outdoor air environments, as NO_2_ accelerates AD-like neurodegenerative diseases. Finally, these results revealed a mechanistic basis for treating, ameliorating, or preventing AD-related outcomes in polluted areas.

## Methods

### Animals

Male C57BL/6 J mice and APPswe/PS1dE9 (APP/PS1) transgenic mice were purchased from Beijing HFK Bioscience Co., Ltd. (Beijing, PRC) and were housed in the animal research room at Shanxi University. The animals were maintained in plastic cages on a 12-h light cycle with standard laboratory food and water. For exposure, C57BL/6 J mice (approximately two months old) were randomly divided into three groups (n = 25 mice per group), and APP/PS1 mice (approximately five months old) were divided into two groups (n = 15 mice per group). Then, the NO_2_-inhaling animals were placed in a dynamic inhalation chamber and exposed to 2.5 or 5.0 mg/m^3^ NO_2_ for 5 h/day for 4 weeks. Accordingly, the control mice were exposed to room air in an identical chamber on the same schedule. The desired concentration was obtained by mixing NO_2_ gas with fresh air at the intake port of the chamber, and the diluted gas was evenly distributed across the whole chamber by two perforated gas radiant plates, with one located in the intake port and the other connected to a gas outlet matched with an aspirator pump. A real-time NO_2_ monitor (Wandi, China) was used to detect the concentration of NO_2_ in the exposure chamber. For the MAGL interference experiments, C57BL/6 J mice (approximately two months old) were divided into three groups (n = 25 mice per group). The interference groups were intraperitoneally (*i.p.*) injected with JZL184 (8 mg/kg bw/2 days) and then were exposed to 5 mg/m^3^ NO_2_ (for 5 h/day for 4 weeks) 1.5 h after injection. The NO_2_ exposure group was *i.p.* injected with the vehicle solution, followed by 5 mg/m^3^ NO_2_ inhalationfor 5 h/day for 4 weeks. Correspondingly, the vehicle control animals received vehicle *i.p.* injection and were exposed to room air. All animal experiments were conducted in accordance with National Institutes of Health Guide for the Care and Use of Laboratory Animals, and were approved by the Institutional Animal Care and Use Committee of Shanxi University.

### Behavioral tests

Morris water maze (MWM) studies with mice have principally been performed to measure hippocampal-dependent spatial-based learning and memory. A circular tank with a diameter of 75 cm H × 100 cm was filled with water rendered opaque by the addition of white, non-toxic paint. A circular platform with a diameter of 15 cm was placed 1 cm below the water surface in the center of a specific quadrant of the tank. The animals were given training sessions using visible and invisible platforms for a period of 8 consecutive days (8 sessions in total). In the non-spatial MWM, the mice received 3 consecutive days of training to find the same platform elevated above the water surface. Then, submerged platform training, which consisted of 5 identical daily sessions with 4 trials per day, was conducted. For each trial, the mice were released from the wall of the tank with their faces in the water. The mice were given 60 s to locate the escape platform. If an animal failed to reach the platform within a fixed period of 60 s, it was gently guided to the location and was allowed to stay on it for 10 s. For each training session, the sequence designed for the animals’ training was determined in a random manner that varied each day so that it was different between the separate sessions for each animal and was different for individual animals. Using an Etho Vision video tracking device (Noldus, Netherlands), we recorded the latency in reaching the target as task performance. The average latencies on each hidden platform training day were recorded and compared. Memory tests were conducted in a probe trial performed 24 h after the last training trail. A probe trial was performed in which the platform was removed from the tank and the animals were allowed to swim for 60 s. The number of target annulus crossovers and the time spent in the target quadrant were measured.

### Immunoblots

Cortical or hippocampal tissue was weighed, homogenized and incubated for 30 min in RIPA buffer containing protease inhibitors. The homogenate was then centrifuged at 13,000 g for 10 min at 4 °C. Protein samples were separated on premade 4 – 12% SDS-PAGE gels and were transferred to PVDF membranes (Bio-Rad). After incubation with primary antibodies, including anti-Aβ_42_ (1:1,000, Invitrogen, USA), anti-BACE1 (1:200, Bioss, China), anti-APP (1:200, Bioss, China), anti-COX-2 (1:200, Cayman, USA), and anti-MAGL (1:200, Cayman, USA), overnight at 4 °C, the membranes were blotted with IR Dye 800 CW-conjugated secondary antibody (1:5000, LiCor Biosciences, USA) at room temperature for 1 h. The densitometry of the bands was scanned and measured using a LI-COR Odyssey Infrared Fluorescent System. The density of each band was quantified using Image-pro Express software, version 6.0 (Media Cybernetics, USA) and was normalized to the corresponding β-actin (1:1000, Cell Signaling, USA) value to account for variations in loading.

### Immunohistochemistry

Immunohistochemical staining was performed to assess the distribution and morphology of amyloid plaques, microglia, and astrocytesin coronally sectioned brain slices. The mice were injected intraperitoneally with pentobarbitone and were subsequently transcardially perfused with heparinized saline and 4% paraformaldehyde in PBS. The tissue was fixed in 4% paraformaldehyde overnight and was transferred to 15% and 30% sucrose solutions in 0.01 M PBS. Dehydration was reached when the tissue sank to the bottom. Series of 40 μm coronal brain sections were prepared with a freezing vibratome and were kept in 0.1 M PBS. Immunostaining was performed on free-floating tissue slices using antibodies specific for Aβ_42_ (1:2,000, Invitrogen, USA), followed by incubation with secondary antibodies (1:5000, LiCor Biosciences, USA) specific to astrocytes (GFAP, 1:2,000, Abcam, USA) and microglia (OX42/CD11b, 1:200, Abcam, USA) and then by incubation with IR Dye 800CW-conjugated secondary antibody (1:5000, LiCor Biosciences, USA). After washing, the sections were incubated with 4′-6-diamidino-2-phenylindole (DAPI), a fluorescent stain that binds strongly to DNA, to label the cell nuclei. The stained sections were captured using fluorescent microscopy (Olympus ix71, Japan) and were analyzed and quantified using Image-pro Express software, version 6.0 (Media Cybernetics, USA).

### Histochemistry

Neurodegeneration was evaluated by Fluoro-Jade C (FJC) stainingaccording to the modified manufacturer’s protocol (Millipore, CA, USA). Cryostat-cutbrain sections were incubated in the dark with FJC (0.0001% solution of FJC dye) dissolved in 0.1% acetic acid for 10 min and then were incubated in DAPI (0.5 μg/mL) for 10 min. Subsequently, the slides were washed 3 times for 1 min each with distilled water, air-dried and coverslipped in the dark. The FJC-stained sections were examined under a fluorescence microscope (Olympus ix71, Japan), and imaging data were analyzed and quantified using Image-pro Express software, version 6.0 (Media Cybernetics, USA).

### Liquid chromatography-tandem mass spectrometry

2-AG and AA were quantified in the brain tissues of C57BL/6 J mice and APP/PS1 transgenic mice using high-performance liquid chromatography coupled with tandem mass spectrometry (LC-MS/MS). The quantification of 2-AG and AA was based on an external standard curve diluted with methanol. All the standards of the analytes were provided by Cayman Chemical (Ann Arbor, MI, USA). The brain tissue was weighed and extracted individually. Frozen samples were placed in extraction solvent containing 2 mL of acetonitrile in a borosilicate glass tube. After rapid homogenization, the sample was centrifuged at 1,500 g for 10 min. The supernatant was collected and evaporated to dryness under a gentle stream of nitrogen. Then, 200 μL of methanol were added to the extract, which was dried under N_2_ gas. Subsequently, the lipid extract was resuspended using 20 μL of methanol, and 5 μL were injected for LC-MS/MS analysis. A Waters UPLC system coupled with a triple quadrupole Quattro Premiere/XE mass spectrometer (Waters, USA) was used for the analysis. Liquid chromatography was performed using a Waters XSelect CSH C18 column 2.1 × 75 mm, 2.5 μm (Waters, USA), with 0.1% formic acid (A) and 100% acetonitrile (B) as a mobile phase. A flow rate of 400 μL/min was used for gradient elution, which started at 50% B, linearly increased to 90% at 2 min and was subsequently reequilibrated at 50% B for a further 2 min after a 4-min hold. The column temperature was 45 °C. The samples were kept at 4 °C in the autosampler until analysis. The targeted LC-MS/MS analysis was performed in multiple reaction monitoring mode (MRM) using the following transitions: the ESI + MRM transition for 2-AG was 379.4 > 287.3, and the ESI- MRM transition for AA was 303.3 > 259.1. The amounts of 2-AG and AA were calculated from the ratios of peak areas of compounds to external standards and by comparing the results with standard curves.

### PGE_2_ assay

PGE_2_ content was measured using the Prostaglandin E_2_ EIA Kit (Cayman, USA). Briefly, 50 mg of cortical tissue were homogenized in 250 μL phosphate-buffered saline (0.1 M phosphate, pH 7.4, containing 1 mM EDTA and 10 μM indomethacin) and then were centrifuged for 10 min at 8,000 g. The supernatant was collected, diluted 1:500 with enzyme immunoassay buffer, and assayed per the protocol in the kit.

### Genechip array

RNA was extracted from approximately 50 mg of hippocampal tissues, and the collected tissues were immediately flash frozen in liquid nitrogen. Total RNA was extracted using TRIZOL Reagent (Life Technologies, USA) according to the manufacturer’s instructions. The RNA integrity number (RIN) was checked using an Agilent Bioanalyzer 2100 (Agilent Technologies, USA) to ensure RNA integration. The RNeasy Mini Kit and RNase-Free DNase Set (Qiagen, Germany) were used for the purification of qualified total RNA. Samples were selected randomly from 4 mice per group and pooled for subsequent microarray analysis. Gene expression analysis was performed using the Agilent one-color Gene Expression Platform. A total of 8 separate arrays from the Agilent mouse 4 × 44k Chip were conducted for the analysis of pooled total RNA samples from the control and NO_2_ treatment groups. The protocol was performed according to the manufacturer’s instructions.

### Gene expression and pathway analysis

Microarray slides were scanned using an Agilent Microarray Scanner (Agilent Technologies, USA) with the default settings (dye channel: green; scan resolution: 5 μm; PMT 100%; 10%; 16 bit). The data were extracted from the raw microarray image files using Feature Extraction Image Analysis software, version 10.7 (Agilent Technologies, USA). Significant changes in gene expression were represented using DIFF Pval (*P* value, *P* < 0.05 was considered statistically significant). In the present study, we evaluated the differentially expressed genes with significance of *P* < 0.01. Because the expressions of several genes were significantly altered in response to NO_2_ exposure, pathway analysis was performed to determine the potential pathways in which the genes showing differential expression were involved. To better understand the underlying biological mechanisms involved, data files from APP/PS1 mice were submitted electronically to the Web-based tool Onto-Tools (http://www.shanghaibiotech.com/sas.html) available on the Shang Hai Biotechnology Corporation website (Shanghai, China). KEGG (Kyoto Encyclopedia of Genes and Genomes) and Biocarta are two knowledge bases for linking genomes to life through the process of pathway mapping. The pathway database consist graphical diagrams of biochemical pathways, including a variety of metabolic and regulatory pathways, our present results presented some specific selected and interested pathways in Fig. 5a.

### Statistical analysis

The data are presented as the means ± S.E. Unless stated otherwise, statistical significance was assessed using one-way ANOVA with Fisher’s PLSD test among groups. Differences for all tests were considered significant when *P* < 0.05.

## Additional Information

**How to cite this article**: Yan, W. *et al.* NO_2_ inhalation promotes Alzheimer's disease-like progression: cyclooxygenase-2-derived prostaglandin E_2_ modulation and monoacylglycerol lipase inhibition-targeted medication. *Sci. Rep.* **6**, 22429; doi: 10.1038/srep22429 (2016).

## Supplementary Material

Supplementary Information

## Figures and Tables

**Table 1:** Information on differentially increased expression genes in AA metabolism pathway.

Gene ID	Symbol	P values	Fold change	Official Full Name
11686	*alox12b*	1.0E-4	1.4025	glutathione peroxidase 7
12408	*cbr1*	2.37E-05	1.2371	carbonyl reductase 1
70101	*cyp4f16*	0.0039	0.8822	cytochrome P450, family 4, subfamily f, polypeptide 16
72303	*cyp2c65*	0.0024	0.6485	cytochrome P450, family 2, subfamily c, polypeptide 65
74519	*cyp2j9*	0.003	0.9182	cytochrome P450, family 2, subfamily j, polypeptide 9
67305	*gpx7*	0.0022	0.8851	glutathione peroxidase 7
66350	*pla2g12a*	0.0005	0.8603	phospholipase A2, group XIIA
19225	*ptgs2*	0.0021	1.2576	prostaglandin-endoperoxide synthase 2
96979	*ptges2*	0.0009	0.8691	prostaglandin E synthase 2
21391	*tbxas1*	0.0087	0.8167	thromboxane A synthase 1, platelet
