# Supplementary material for: NO2 inhalation promotes Alzheimer’s disease-like progression: cyclooxygenase-2-derived prostaglandin E2 modulation and monoacylglycerol lipase inhibition-targeted medication
Source: Sci Rep. 2016 Mar 1;6:22429. doi: 10.1038/srep22429 (PMC4772479; doi:10.1038/srep22429)
Supplement: Supplementary Information [file srep22429-s1.doc]

**Supplementary Information**

**NO_2_ inhalation promotes Alzheimer’s disease-like progression: cyclooxygenase-2-derived prostaglandin E_2_ modulation and monoacylglycerol lipase inhibition-targeted medication**

Wei Yan^1^, Yang Yun^1^, Tingting Ku, Guangke Li, Nan Sang*

*College of Environment and Resources, Research Center of Environment and Health, Shanxi University, Taiyuan, Shanxi 030006, PR China*

***^1^*** These authors contributed equally to this work.

****Corresponding author:*** Nan Sang, College of Environment and Resource, Shanxi University, Taiyuan, Shanxi Province, China. Tel.: 86-351-7011932; Fax: 86-351-7011932; E-mail: [sangnan@sxu.edu.cn](mailto:sangnan@sxu.edu.cn).

**
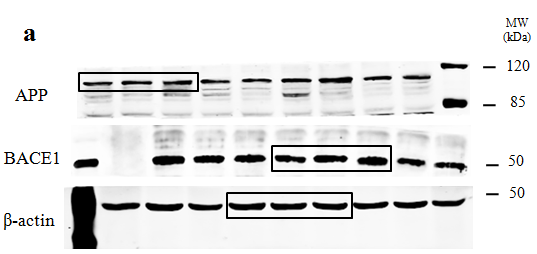
**


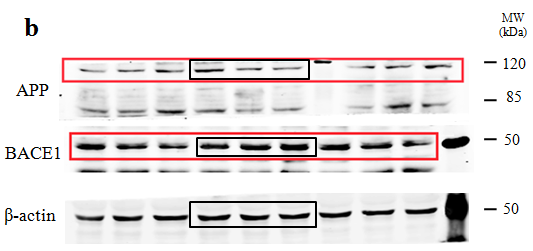


Figure S1. Presentation of original immunoblot shown in Figure 2a and b. All the gels were run under the same experimental conditions. In the same gel, a prestained protein molecular weight marker (Thermo Scientific, USA) was used to identify the target bands. The red boxes indicated the target proteins when multiple bands were shown on the electrophoresis gel. The cropped parts of immunoblot presented in Figure 2 were indicated with black boxes.


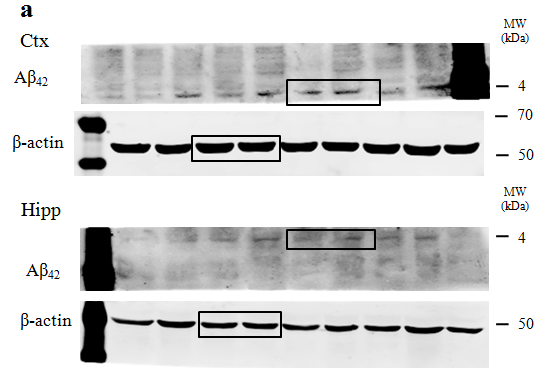


Figure S2. Presentation of original immunoblot shown in Figure 4a. All the gels were run under the same experimental conditions. In the same gel, a prestained protein molecular weight marker (Thermo Scientific, USA) was used to identify the target bands. The cropped parts of immunoblot presented in Figure 4 were indicated with black boxes.


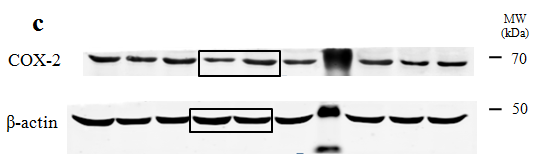


Figure S3. Presentation of original immunoblot shown in Figure 6c. All the gels were run under the same experimental conditions. In the same gel, a prestained protein molecular weight marker (Thermo Scientific, USA) was used to identify the target bands. The cropped parts of immunoblot presented in Figure 6 were indicated with black boxes.


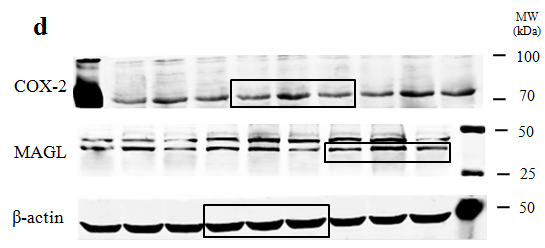


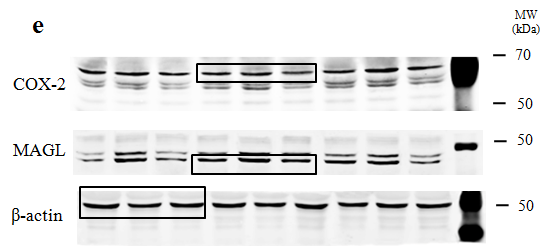


Figure S4. Presentation of original immunoblot shown in Figure 7d and e. All the gels were run under the same experimental conditions. In the same gel, a prestained protein molecular weight marker (Thermo Scientific, USA) was used to identify the target bands. The cropped parts of immunoblot presented in Figure 7 were indicated with black boxes.


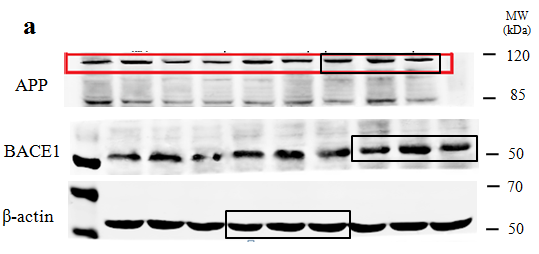


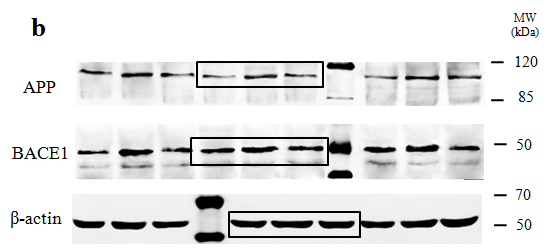


Figure S5. Presentation of original immunoblot shown in Figure 8a and b. All the gels were run under the same experimental conditions. In the same gel, a prestained protein molecular weight marker (Thermo Scientific, USA) was used to identify the target bands. The red boxes indicated the target proteins when multiple bands were shown on the electrophoresis gel. The cropped parts of immunoblot presented in Figure 8 were indicated with black boxes.
